# Prenatal bisphenol A and/or diethylhexyl phthalate exposure followed by adult estradiol treatment affects behavior and brain monoamines in female rat offspring

**DOI:** 10.3389/fendo.2024.1479838

**Published:** 2025-01-06

**Authors:** Amrita Kaimal, Jessica M. Hooversmith, Maryam H. Al Mansi, Ariana D. Cherry, Jillian T. Garrity, Philip V. Holmes, Puliyur S. MohanKumar, Sheba M. J. MohanKumar

**Affiliations:** ^1^ Biomedical and Translational Sciences Institute, Neuroscience Division, Athens, GA, United States; ^2^ Department of Biomedical Sciences, College of Veterinary Medicine, University of Georgia, Athens, GA, United States

**Keywords:** bisphenol A (BPA), diethylhexyl phthalate (DEHP), prenatal exposure, behavior, monoamine neurotransmitters, endocrine-disrupting chemical (EDC) mixtures

## Abstract

**Significance:**

Women are at increased risk for mood disorders, which may be partly attributed to exposure to endocrine-disrupting chemicals (EDCs) during sensitive periods such as pregnancy. Exposure during these times can impact brain development in the offspring, potentially leading to mood disorders in later life. Additionally, fluctuating levels of endogenous estrogens, as seen during pregnancy, or the use of oral contraceptives, can further elevate this risk. This study examines the cumulative effects of prenatal EDC exposure combined with chronic low-dose 17β-estradiol (E2) treatment in adulthood on neurobehavioral outcomes.

**Methods:**

Pregnant Sprague-Dawley rats were orally dosed with vehicle, bisphenol A (BPA) (5 μg/kg body weight (BW)/day), low-dose (LD) diethylhexyl phthalate (DEHP) (5 μg/kg BW/day), high-dose (HD) DEHP (7.5 mg/kg BW/day), or a combination of the two (BPA+DEHP) from gestational days 6-21. At 3 months of age, female offspring were implanted with slow-release E2 pellets or were sham-implanted. Following a 90-day treatment period, behavioral testing was conducted, and serum hormones and brain monoamine levels were analyzed.

**Results:**

Chronic E2 treatment in controls increased anxiety and reduced active coping behaviors. In DEHP- and BPA+DEHP-exposed offspring, E2 treatment reversed some of these effects. Dose-dependent alterations in circulating hormone levels and brain monoamines were observed. Dysregulation of the stress axis was particularly notable with the higher dose of DEHP.

**Conclusions:**

Overall, prenatal EDC exposure altered behavior, hormones, and brain monoamines, with adult E2 treatment further exacerbating some of these effects in female offspring.

## Introduction

Women are at a higher risk for developing mood disorders (1, 2). This increased vulnerability may stem from exposure to endogenous estrogens (3), environmental estrogens (4) and endocrine-disrupting chemicals (5). Early life exposure to EDCs such as bisphenol-A (BPA) and diethyl hexyl phthalate (DEHP) poses a particular threat to women, as these chemicals can mimic or disrupt estrogen’s natural actions (6, 7). Pregnant women who use cosmetics containing phthalates (8) or are exposed to BPA and phthalates commonly found in the environment- through inhalation, ingestion or skin contact- risk passing these chemicals to their fetuses (9, 10). These EDCs can cross the placental barrier potentially causing long-term neurobehavioral effects in the developing fetus (11–14). Even more concerning is the potential for exposure to combinations of these EDCs which may have a greater harmful impact than exposure to individual EDCs alone (15, 16).

Prenatal exposure to these EDCs is thought to produce subtle changes in the developing brain (17), which may later manifest as mood disorders in adulthood. The underlying mechanisms remain poorly understood. Specific brain regions, such as the cortex and hippocampus, are likely implicated in the onset of these mood disorders (18). Changes in neurotransmitter levels, particularly, norepinephrine (NE), dopamine (DA) and serotonin (5-HT)- which have long been associated with mood disorders (19, 20)- may also play a role. However, the impact of prenatal EDC exposure on neurotransmitter levels in specific brain regions has yet to be thoroughly investigated.

In addition to neurotransmitter alterations, fluctuations in estrogen levels are known to contribute to mood disorders (20). Sensitive periods, such as the menstrual cycle and pregnancy are associated with fluctuating estrogen levels (21). Women are also exposed to estrogens through oral contraceptives and hormone replacement therapy, both of which have been linked to mood disorders in some women (22). In previous studies using a rodent model, we demonstrated that implanting a slow-release pellet delivering 20 ng of estradiol per day over 90 days induced anxiety-like behavior, which was associated with reduced dopamine levels in the amygdala (23). This dose of estradiol produces circulating estrogen levels that are twice the levels seen in rats on the day of proestrus (24) and may be comparable to that in pre-menopausal women. In the current study, we aim to investigate whether prenatal exposure to EDCs, followed by chronic low-dose estradiol exposure to mimic oral contraceptive therapy, increases the risk of mood disorders in female offspring.

To achieve this, we exposed pregnant animals to low doses of BPA and DEHP, either individually or in combination, and then subjected their adult female offspring to chronic low-dose E2 exposure. A battery of behavioral tests was conducted to evaluate various behavioral outcomes, which were then correlated with changes in hormone and neurotransmitter levels.

The BPA dose was selected because it is significantly lower than the Environmental Protection Agency (EPA) recommended no-observed-adverse-effect-level (NOAEL) dose of 5 mg/kg/day (25), as well as 10-fold below the tolerable daily intake (TDI) dose of 50 µg/kg/day (26). Additionally, this dose is within the estimated range of BPA exposure in humans (0.4-5 µg/kg/day) (27). The high dose of DEHP used in our study is higher than the established NOAEL dose of 4.8 mg/kg/day (28), whereas the low DEHP dose is significantly lower than this. Additionally, the low dose of DEHP used lies within the range of the typical daily intake of DEHP in adult humans (0.5-25 µg/kg/day) (29), and is well below the EPA reference dose of 20 µg/kg/day (30).

## Materials and methods

### Animals

Adult female Sprague-Dawley rats purchased from Envigo (Indianapolis, IN) were housed in light- (12:12 light-dark cycle) and temperature-controlled (23.2 ± 2°C, 50 ± 20% relative humidity) animal rooms at the University of Georgia, with food and water provided *ad libitum*. The rats were fed Pico Lab Rodent Diet 20 (LabDiet) and housed in polycarbonate cages with corn cob bedding. The female breeders underwent vaginal cytology for 10 consecutive days prior to mating to track their individual estrous cycles. Females in proestrus were randomly assigned a male and the two were co-housed for one day. The presence of a vaginal plug was used to confirm the occurrence of mating. Gestational day (GD) 0 represented the day of copulation. Experimental protocols followed the National Institutes of Health’s *Guide for the Care and Use of Laboratory Animals* approved by the Institutional Animal Care and Use Committee (IACUC) at the University of Georgia.

### Chemicals

BPA (Lot MKBH2096V; Catalog No. 239658; Purity: ≥ 99.0%) and DEHP (Lot BCBR8079V; Catalog No. 36735; Purity: ≥ 98.0%) were purchased from Sigma Aldrich (St. Louis, MO). BPA has been studied extensively; therefore, we incorporated it as a positive control in this study and only tested the effects of a single low dose.

### EDC and E2 exposure paradigms

**Figure 1:**
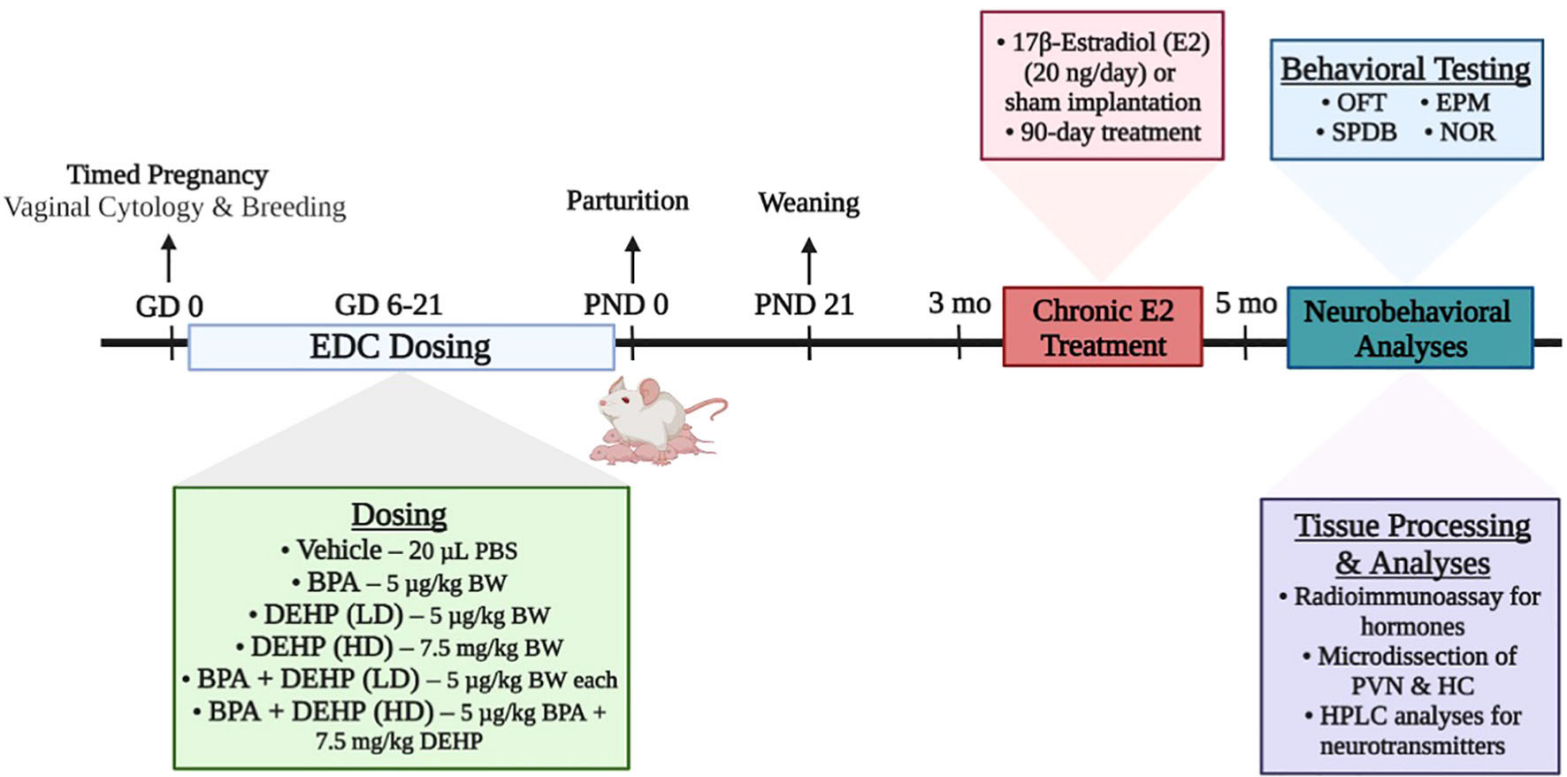
Summary of the experimental design of the study. Pregnant Sprague-Dawley dams were orally dosed daily from gestational days (GD) 6-21 with vehicle (Control) (20 µL PBS; *n*=7), BPA (5 µg/kg/day; *n*=9), low-dose (LD) DEHP (5 µg/kg/day; *n*=6), high-dose (HD) DEHP (7.5 mg/kg/day; *n*=6), a mixture of BPA + LD DEHP (5 µg/kg/day of BPA + 5 µg/kg/day of DEHP; n=6), or a mixture of BPA + HD DEHP (5 µg/kg/day of BPA + 7.5 mg/kg/day of DEHP; n=7). Adult female offspring aged 3 months were either sham-implanted or implanted with a slow release 90-day E2 pellet. Approximately 60-70 days into the treatment, each offspring underwent behavioral testing once and were euthanized immediately after. Trunk blood was collected for the measurement of serum hormones using radioimmunoassay. Brains were micro-dissected for PVN and HC tissues, which were then analyzed for monoamines and major metabolites using HPLC. Experimental design schematic was created using Biorender.com. EDC, endocrine-disrupting chemical; PBS, Phosphate Buffered Saline; BPA, bisphenol A; DEHP, diethylhexyl phthalate; LD, low-dose; HD, high-dose; OFT, open field test; EPM, elevated plus maze; SPDB, shock probe defensive burying; NOR, novel object recognition test; PVN, paraventricular nucleus; HC, hippocampus; HPLC, high-performance liquid chromatography.

The experimental design is demonstrated in **Figure 1**. Stock solutions were made in dimethylsulfoxide (DMSO; 1 µg/µl for BPA and low dose DEHP and 1 mg/µl for high dose DEHP). Doses were calculated daily based on body weight and the volumes ranged from 1-3 µl. Control rats received DMSO based on their body weight. Volume measured for each rat was mixed with 20 µl Phosphate Buffered Saline (PBS) for oral dosing. Daily oral dosing occurred from GD 6-21 since organogenesis in the fetus occurs around GD 9-12. The vehicle or EDC treatments were discharged into the oral cavity using a micropipette to avoid any local irritation to the gastrointestinal tract and potential stress to the pregnant dam. The dam was considered the experimental unit. Each dam was randomly assigned to one of 6 different treatment groups: control (n=7), BPA (5 µg/kg BW/day; n=9), low-dose (LD) DEHP (5 µg/kg BW/day; n=6), high-dose (HD) DEHP (7.5 mg/kg BW/day; n=6), a combination of BPA and LD-DEHP (5 µg/kg/day of BPA + 5 µg/kg/day of DEHP; n=6), and a combination of BPA and HD-DEHP (5 µg/kg/day of BPA + 7.5 mg/kg/day of DEHP; n=7).

Once the female offspring of these dams reached 3 months of age, vaginal cytology was used to determine estrous cyclicity, and those with regular estrous cycles were included in the experiments. Approximately two female offspring from each dam were used; one was sham-implanted (control) (n=6-7/group), and the other was implanted with a slow release 17β-Estradiol (E2) pellet (n=6-9/group) (1.8 µg, Innovative Research America, Sarasota, FL). The E2 pellets release 20 ng per day over 90 days, which leads to constant estrus in rats after 60 days of exposure as a result of accelerated reproductive aging (31, 32). Therefore, control offspring underwent behavioral testing only when they were in estrus.

### Behavioral testing

The adult female offspring of the treated dams were transferred to another facility on campus two weeks prior to behavioral testing, where they remained undisturbed during this habituation period. Animals were group housed (2-4 rats per cage) with rats of the same dose group in polycarbonate cages with corn cob bedding. Rooms were maintained at 23.3 ± 3°C on a 12:12 reverse light-dark cycle. All behavioral testing occurred during the dark cycle. All animals had access to food and water *ad libitum* in their home cages, including before and after each testing session.

The sham- and E2-treated female offspring were administered a battery of behavioral tests approximately 60-70 days into their treatments and all offspring were tested and euthanized by the end of their 90-day treatments. The behavioral paradigms employed were the Open Field Test (OFT), Elevated Plus Maze (EPM), and Shock Probe Defensive Burying (SPDB). The Novel Object Recognition test (NOR) was also administered, but only to the animals in the LD group since these offspring demonstrated more intriguing behavioral effects and we wanted to examine how their cognition was affected as a result. The order for the tests was OFT, EPM, SPDB, followed by NOR.

Testing was done after the lights were turned off. The open field test was run for 10 minutes, EPM was 5 minutes, and SPDB was 10 minutes. The animal was allowed to acclimate to each testing room for 5 minutes prior to testing and data from the entire period (or the first 10 minutes) was included in the analysis. The NOR test was for a total of 53 minutes: 1) a 5-minute familiarization phase, 2) a 45-minute retention phase, and 3) a 3- minute test phase. The tests were administered in succession and each rat was exposed to each test only once. Rats were then euthanized after completion of the behavioral testing. Both testing and euthanasia for each rat occurred on the same day. Vaginal smears were obtained from all rats, for 2-10 days prior to behavioral testing to ensure that animals were all tested in estrus. The behavioral tests were conducted exactly as described in Kaimal et al. (33). A brief description of each of these tests is provided below:

#### Open field test

Each animal was placed in a transparent plexiglass test chamber measuring 43.3 cm in length and width and 30.5 cm in height, divided into a center zone and a perimeter zone, as described earlier (34, 35). The perimeter zone encompassed the area within 0-9 inches from the walls, while the center zone extended from 9-35 inches from the walls. At the start of each session, rats were positioned in the lower left corner of the chamber, facing the opposite wall, and allowed to explore freely for 10 minutes. Their movements were recorded using Activity Monitor software (Med Associates, Fairfax, VT, USA) on a desktop computer, which automated the tracking process and provided unbiased data analysis. Key measures recorded during the test included the number of entries and time spent in the center and perimeter zones, the frequency and duration of rearing behavior, as well as total distance traveled and time spent ambulating within the chamber.

#### Elevated plus maze

The EPM apparatus consisted of a wooden maze painted in matte black, featuring two pairs of arms set perpendicular to each other and elevated 50 cm above the floor. The setup included two open arms (45 x 9 cm) without walls and two closed arms (45 x 9 x 38 cm) enclosed by high walls but without a ceiling. To start the test, the animal was placed on the central platform (9 x 9 cm), facing an open arm opposite the experimenter. During the session, the number of entries and time spent in each arm, as well as crossings through the central platform, were recorded. An entry into an arm was defined as the animal having all four feet within the arm.

#### Shock probe defensive burying test

This behavioral assessment followed the protocol outlined by Kaimal et al. (33). Animals were subjected to a mild shock of 3 mA DC, as previously described (34). Testing was conducted during the dark phase of the light cycle, with animals exposed to red lighting. An overhead webcam (Microsoft) was used to record the animals’ responses. Typically, animals react to the stimulus by attempting to bury the probe. The duration of this burying behavior was measured over a 10-minute testing period, with all recordings manually scored using a double-blind method. This test effectively reveals defensive behaviors in animals and provides insight into the coping mechanisms employed by the subjects.

#### Novel object recognition test

This test was conducted as previously described by Kaimal et al. (33). Briefly, rats were placed in test chambers made of Sterlite that had no ceilings and opaque walls. Objects of varying shapes and sizes that were previously tested for object preference bias were glued to a small jar that was attached to the box (34). For the familiarization phase of the test, animals were allowed to explore two identical objects for 5 minutes after which animals were returned to their home cages for 45 minutes. During the test phase, the animals were brought back to the test chamber that now contained one of the previously explored objects and a novel object. The animal was allowed to explore the familiar and novel objects for 3 minutes. The difference in exploration time of the novel and familiar object divided by the total exploration time was used as the discrimination index (DI). The recognition index (RI) is calculated as the percent time spent exploring the novel object relative to the total time spent exploring both objects.

### Tissue collection and preparation

Female offspring were euthanized by rapid decapitation immediately after behavioral testing. Brains were dissected, and trunk blood was collected and centrifuged. Brain and serum samples were stored at -80°C for further processing.

### Hormone measurement

Serum estradiol (E2), corticosterone (CORT), and oxytocin (OXT) levels were measured in duplicate using a double antibody radioimmunoassay (E2 & CORT – MP Biomedicals, Santa Ana, CA; E2 SKU: 0713810-CF; CORT SKU: 07120121; OXT – Phoenix Pharmaceuticals, Burlingame, CA; Catalog No. RK-051-01), according to the manufacturer’s protocol. CORT values were expressed as ng/ml. E2 and OXT values were expressed as pg/ml.

### Brain sectioning and microdissection

A cryostat (Slee, London, UK) maintained at -10°C was used to section brains at 300 µm thickness. Following this, the paraventricular nucleus of the hypothalamus (PVN) and ventral subdivision of the HC were micro-dissected on a cold stage using the Palkovits’ microdissection technique and a stereotaxis brain atlas as a reference (36). All brain punches were obtained using a 500µm punch (Zivic instruments, Pittsburgh, PA) and stored at -80°C until further analyses. Care was taken to ensure that the sections corresponded to the following co-ordinates: PVN: 1.8-2.1mm posterior, 0-0.3 mm lateral and 7-8 mm ventral to the Bregma; HC: 3-6 mm posterior, 0-5mm lateral, 3-8mm ventral to the Bregma. Subdivisions of these nuclei were also included in the punches.

### Neurotransmitter analysis by HPLC-EC

HPLC-EC was used to analyze brain punches for NE, DA, DOPAC, 5-HT, and 5-HIAA as previously described in (37) and in Kaimal et al., 2023 (33). Brain punches were briefly homogenized in 0.05 M perchloric acid on ice and an aliquot was used for protein estimation (MicroBCA assay, Pierce, Rockford, IL). The remaining homogenate was centrifuged at 18,000 × g for 8 min at 4°C. The supernatant was injected with an internal standard (dihydroxybenzylamine, 0.05 M) into the autoinjector for HPLC analysis. Chromatograms were analyzed for neurotransmitter concentrations using the Class VP software v 7.2 (Shimadzu, Columbia, MD). Neurotransmitter concentrations in tissue samples were expressed as pg/µg of protein. Protein levels in tissue punches were measured using the micro bicinchoninic acid assay (Pierce, Rockford, IL). Samples were assayed in duplicate according to the manufacturer’s protocol. Besides actual neurotransmitter values, turnover rates for DA and 5-HT were obtained by dividing the concentrations of the metabolites by the concentration of the parent neurotransmitter.

### Statistical analysis

Prism 9.0.0 (GraphPad, Inc.) software was used to perform statistical analyses. All data were analyzed by two-way ANOVA (EDC exposure × sex) with behavioral parameters, hormones, or neurotransmitters as the dependent variables. Interaction effects between EDC exposure and sex were also assessed. Differences in behavioral parameters between control and EDC groups, as well as between sham and E2 groups, were analyzed using 2-way ANOVA followed by Fisher’s LSD *post-hoc* test. EDC effects in hormonal and neurotransmitter data and the differences between Sham and E2-implanted animals were analyzed using 2-way ANOVA followed by Tukey’s multiple comparisons test. P-value < 0.05 was considered to indicate a statistically significant difference. Data is expressed as mean ± standard error of mean (SEM).

## Results

### Behavioral effects

#### Open field test

**Figure 2:**
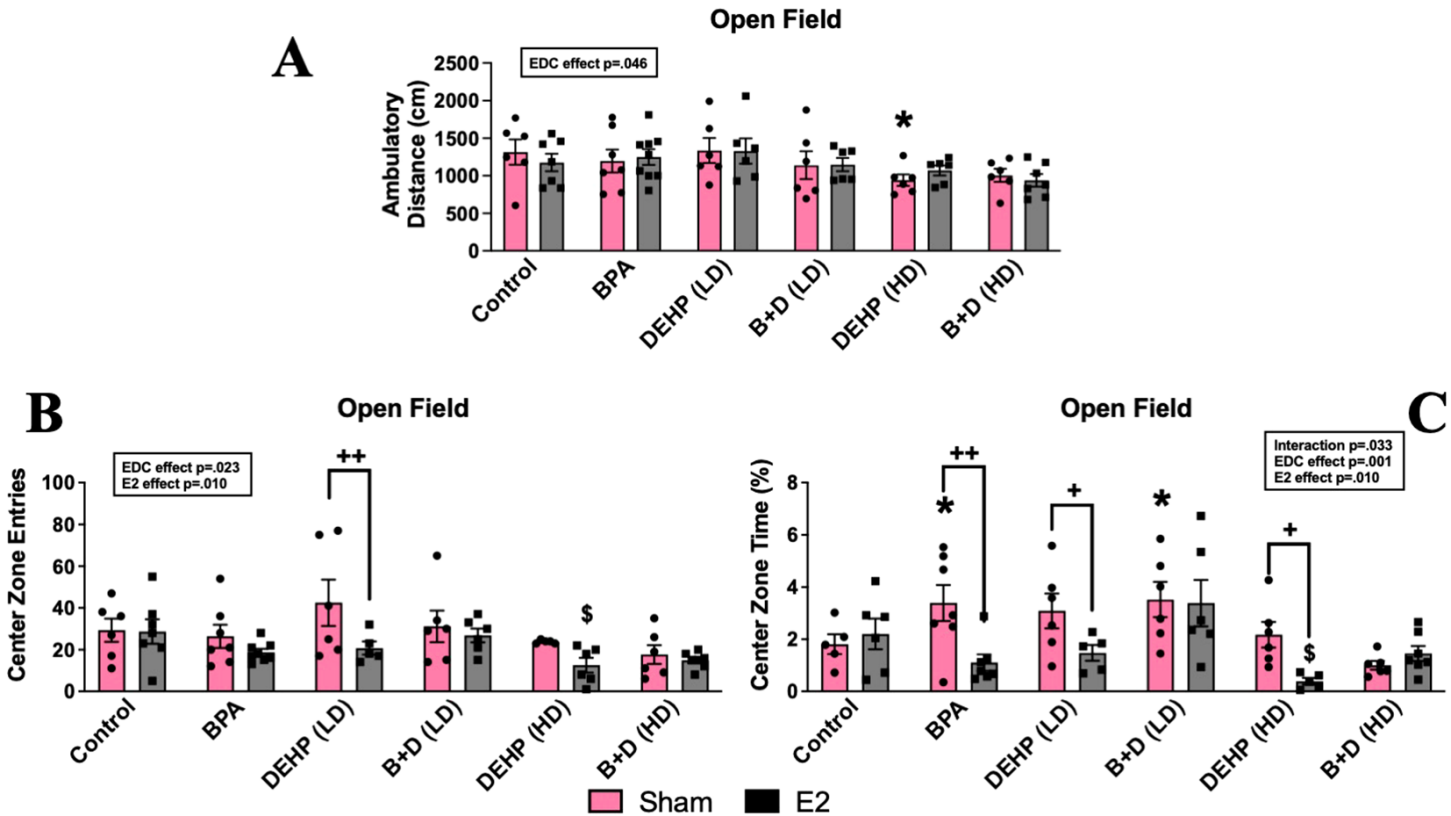
Behavioral effects of prenatal EDC exposure and/or adult E2 treatment in female rat offspring in the open field test (OFT). **(A)** Locomotor activity, **(B)** center zone time, and **(C)** center zone entries. Data were collected from sham- or E2-implanted adult female offspring prenatally exposed to vehicle (Control) (sham: *n*=5-6; E2: *n*=6-7), BPA (sham: *n*=7; E2: *n*=7-9), DEHP (LD) (sham: *n*=6; E2: *n*=5-6), a mixture of BPA + DEHP (LD) (sham: *n*=6; E2: *n*=6), DEHP (HD) (sham: *n*=5-6; E2: *n*=5-6), or a mixture of BPA + DEHP (HD) (sham: *n*=6; E2: *n*=7). Data were analyzed by two-way ANOVA, followed by Fisher’s LSD *post hoc* test. ^*^ *p*<0.05, comparison between sham-implanted control and EDC-exposed female offspring. ^$^ *p*<0.05, comparison between E2-implanted control and EDC-exposed female offspring. ^+^ *p*<0.05, ^++^p<0.01, comparison between sham- and E2-implanted female offspring of the same EDC treatment group. Error bars represent the standard error of the mean (SEM).

A modest EDC exposure effect (*p* = 0.046; F (5, 66) = 2.4) was observed in ambulatory distance (cm, mean ± SEM) (**Figure 2A**), wherein DEHP (HD)-sham offspring (941.3 ± 76.0; *p* = 0.049) traveled less within the chamber relative to their control counterparts (1316.8 ± 167.5). Although DEHP (HD) exposure reduced ambulation in sham offspring in this test, these offspring did not display changes in activity levels in the EPM. Therefore, there is no clear effect on locomotor activity in these females. No significant differences were observed in the amount of time spent ambulating or rearing within the chamber in any of the groups (**Table 1**).

**Table 1:** Behavioral data of sham and E2-treated female offspring following low-dose (5 µg) and high-dose (7.5 mg) prenatal EDC exposure.

Measure	Control		BPA (5 µg)		DEHP (5 µg)		BPA + 5 µg DEHP		DEHP (7.5 mg)		BPA + 7.5 mg DEHP	
	Sham	E2	Sham	E2	Sham	E2	Sham	E2	Sham	E2	Sham	E2
OFT												
*Ambulation (% time)*	21.6 ± 2.8	17.8 ± 1.8	17.6 ± 2.4	18.5 ± 1.9	22.5 ± 2.7	18.0 ± 1.2	18.2 ± 3.4	17.3 ± 1.1	15.0 ± 1.3	16.4 ± 1.2	16.4 ± 1.6	15.7 ± 1.6
*Rearing (frequency)*	82.7 ± 14.9	86.0 ± 10.0	69.2 ± 1.4	72.5 ± 4.0	80.2 ± 4.7	87.8 ± 10.7	91.7 ± 10.7	66.4 ± 3.9	114.0 ± 29.7	61.2 ± 2.5	83.2 ± 6.7	70.3 ± 9.2
*Rearing (% time)*	21.6 ± 4.4	27.4 ± 4.8	25.6 ± 4.7	18.8 ± 2.0	24.0 ± 2.7	25.2 ± 3.4	24.7 ± 2.5	23.5 ± 2.4	23.1 ± 2.2	19.6 ± 2.1	16.4 ± 1.4	17.5 ± 2.1
*Perimeter zone (entries)*	92.5 ± 12.9	78.6 ± 9.9	86.3 ± 7.5	92.1 ± 11.8	61.3 ± 4.1	87.7 ± 2.5	71.3 ± 12.6	72.3 ± 5.9	52.3 ± 8.1	76.8 ± 9.4	62.0 ± 6.2	75.6 ± 11.7
*Perimeter zone (% time)*	39.6 ± 6.5	33.4 ± 5.5	36.9 ± 4.8	41.1 ± 4.6	26.9 ± 6.2	39.7 ± 6.2	36.6 ± 2.5	38.4 ± 1.0	32.2 ± 8.8	37.8 ± 5.7	42.8 ± 6.2	49.4 ± 4.9
EPM												
*Central platform (% time)*	22.9 ± 4.6	11.9 ± 1.9	21.2 ± 3.6	17.5 ± 1.9	10.3 ± 1.5^**^	10.5 ± 1.2	10.8 ± 2.2^**^	13.4 ± 2.2	16.7 ± 4.7	17.2 ± 3.6	18.8 ± 2.4	21.2 ± 3.0**^$^**
*Open arms (% time)*	42.6 ± 5.4	27.5 ± 5.3	53.6 ± 7.8	45.3 ± 6.9	48.5 ± 8.6	57.2 ± 10.2	10.8 ± 2.3	39.3 ± 7.7	33.6 ± 5.1	44.4 ± 7.1	39.7 ± 6.9	46.8 ± 3.2
*Closed arms (# of entries)*	8.8 ± 1.0	10.8 ± 0.5	7.4 ± 1.6	10.0 ± 1.6	11.5 ± 1.8	9.3 ± 2.2	10.8 ± 2.4	10.5 ± 0.8	12.0 ± 0.8	9.7 ± 0.9	12.0 ± 2.1	9.0 ± 1.0
*Closed arms (% time)*	34.0 ± 8.0	60.0 ± 6.3	24.8 ± 5.7	36.9 ± 6.6	41.0 ± 7.4	32.1 ± 10.9	10.8 ± 2.5	46.7 ± 8.6	41.4 ± 7.5	38.1 ± 5.8	40.7 ± 7.3	31.6 ± 3.4
SPDB												
*Burying (frequency)*	25.2 ± 6.8**^+^**	7.3 ± 3.1**^+^**	9.7 ± 3.2	6.8 ± 1.7	7.0 ± 3.5^*^	18.2 ± 8.7	14.2 ± 7.2**^a^**	30.7 ± 10.1**^$$,a^**	21.2 ± 7.5	6.2 ± 2.0	10.3 ± 5.4	12.9 ± 5.0
*Immobility (frequency)*	12.7 ± 5.8	16.7 ± 2.2	17.7 ± 4.8	11.1 ± 1.4	24.7 ± 5.1^*^	24.8 ± 3.3	19.5 ± 4.8	12.8 ± 3.2	10.8 ± 4.5	9.2 ± 2.7	9.8 ± 4.0	8.0 ± 1.6
*Probe exploration (frequency)*	14.3 ± 4.4	10.1 ± 4.3	13.4 ± 3.1	12.1 ± 2.5	13.3 ± 4.9	4.5 ± 1.1	9.8 ± 3.0	9.2 ± 4.1	8.2 ± 2.3	5.3 ± 2.4	9.2 ± 2.1	9.1 ± 3.0
*Rearing (frequency)*	19.3 ± 4.8	12.1 ± 5.4	20.6 ± 4.3	17.3 ± 3.5	14.8 ± 3.9	8.0 ± 2.7	13.5 ± 5.5	12.7 ± 4.0	15.8 ± 4.0	8.8 ± 2.0	17.7 ± 4.0	15.2 ± 3.3
*Grooming (frequency)*	0.0 ± 0.0	0.0 ± 0.0	0.4 ± 0.3	0.6 ± 0.4	0.5 ± 0.3	0.2 ± 0.2	0.5 ± 0.3	0.0 ± 0.0	0.0 ± 0.0	0.7 ± 0.4	0.2 ± 0.2	0.6 ± 0.4
*Grooming (% time)*	0.0 ± 0.0	0.0 ± 0.0	0.5 ± 0.3	1.4 ± 0.9	0.7 ± 0.6	0.9 ± 0.9	0.5 ± 0.3	0.0 ± 0.0	0.0 ± 0.0	1.2 ± 0.9	0.1 ± 0.1	0.5 ± 0.4
*Bedding height (cm)*	7.8 ± 0.7	7.4 ± 0.6	6.3 ± 0.6	6.1 ± 0.3	5.9 ± 0.4	7.1 ± 0.8	6.8 ± 0.6	8.4 ± 0.6	6.4 ± 0.6	6.1 ± 0.4	6.3 ± 0.5	6.4 ± 0.6
*Shock reactivity*	1.7 ± 0.2	1.3 ± 0.2	1.1 ± 0.1	1.1 ± 0.1	1.0 ± 0.0	1.3 ± 0.2	1.0 ± 0.0	1.2 ± 0.2	1.3 ± 0.2	1.0 ± 0.0	1.0 ± 0.0	1.4 ± 0.2
NOR												
*T1 Average Exploration (% time)*	19.6 ± 4.6	21.3 ± 4.3	20.9 ± 1.2	19.4 ± 1.5	22.0 ± 1.6	20.0 ± 2.6	24.6 ± 2.5	15.1 ± 2.3	N/A	N/A	N/A	N/A
*T2 Discrimination Index*	0.01 ± 0.1	0.3 ± 0.1	0.2 ± 0.1	0.3 ± 0.1	0.2 ± 0.1	0.2 ± 0.1	0.2 ± 0.1	0.1 ± 0.2	N/A	N/A	N/A	N/A

EDC, endocrine-disrupting chemicals; E2, estradiol; BPA, bisphenol A; DEHP, di-(2-ethylhexyl) phthalate; OFT, open field test; EPM, elevated plus maze; SPDB, shock probe defensive burying; NOR, novel object recognition test. Data are presented as mean ± SEM. Data were analyzed using two-way ANOVA, followed by Fisher's LSD post hoc analyses. ^*^ p < 0.05, ^**^ p < 0.01, significantly different from Control-Sham group. **^$^** p < 0.05; **^$$^** p < 0.01, significantly different from Control-E2 group. **^+^** p < 0.05, significant difference between Sham and E2-treated Control offspring. **^a^** p = 0.05, difference between Sham and E2-treated BPA + 5µg DEHP offspring.

N/A, not applicable.

Significant main effects of EDC (*p* = 0.001; F(5,60)=4.7) and E2 (*p* = 0.01; F(1,60)=7.2), as well as an interaction effect (*p* = 0.033; F(5,60)=2.6), were found in time spent within the center zone (%, mean ± SEM) (**Figure 2B**). Sham-treated BPA (3.4 ± 0.7; *p* = 0.044) and B+D (LD) (3.5 ± 0.7; *p* = 0.035) offspring resembled each other because they demonstrated decreased anxiety-like behavior with robust increases in center time compared to their control counterparts (1.8 ± 0.4). On the contrary, E2-treated offspring exposed to BPA (1.1 ± 0.3; *p* = 0.002), DEHP (LD) (1.5 ± 0.3; *p* = 0.046), and DEHP (HD) (0.4 ± 0.1; *p* = 0.028) displayed anxiogenic effects by having drastic reductions in center time than their sham counterparts. This decrease in center time was present in DEHP (HD)-E2 offspring when compared to control-E2 offspring (2.2 ± 0.6; *p* = 0.025) as well, displaying an interaction between EDC and E2 in DEHP (HD)-E2 females in particular. In terms of center zone entries (mean ± SEM) (**Figure 2C**), the main effects of EDC (*p* = 0.023; F(5,63)=2.8) and E2 (*p* = 0.01; F(1,63)=7) were observed. E2-treated females exposed to DEHP (HD) (12.5 ± 3.5) had significantly lower number of entries into the center zone compared to the control-E2 group (28.7 ± 5.8; p=0.03). No significant differences were observed in time spent or entries into the perimeter zone (**Table 1**).

#### Elevated plus maze

**Figure 3:**
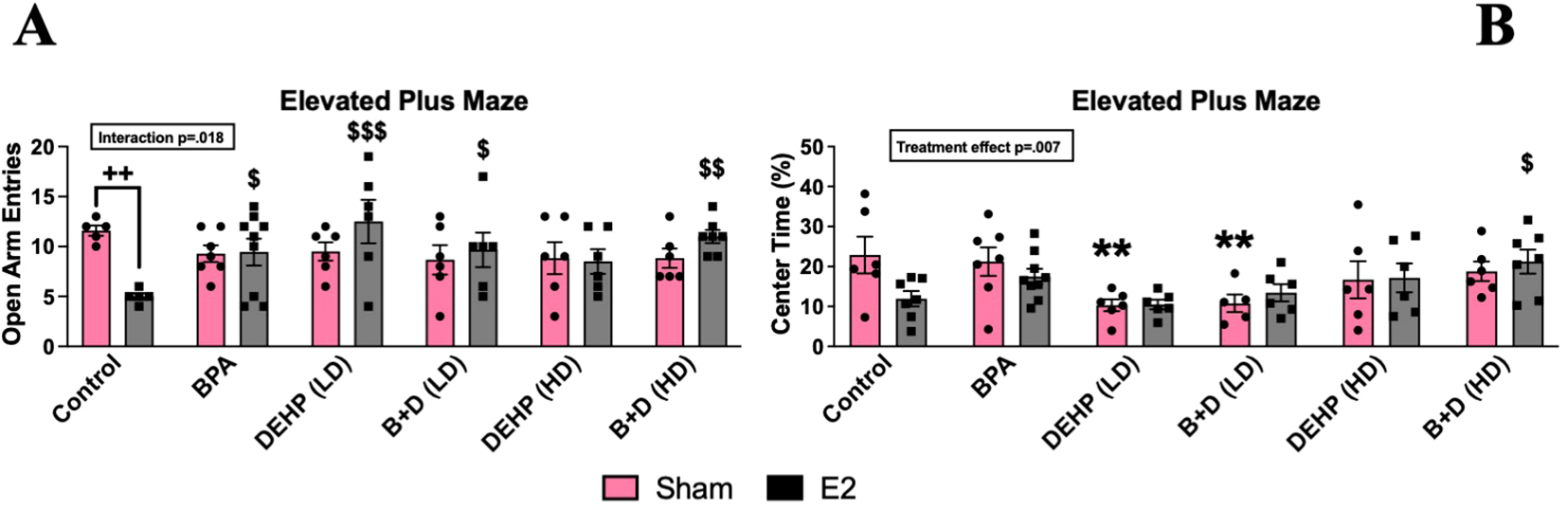
Behavioral effects of prenatal EDC exposure and/or adult E2 treatment in female rat offspring in the elevated plus maze (EPM). **(A)** Number of entries into the open arms and **(B)** percentage of time spent in the center zone. Data were collected from sham- or E2-implanted adult female offspring prenatally exposed to vehicle (Control) (sham: *n*=5-6; E2: *n*=5-7), BPA (sham: *n*=7; E2: *n*=7-9), DEHP (LD) (sham: *n*=5-6; E2: *n*=6), a mixture of BPA + DEHP (LD) (sham: *n*=6; E2: *n*=6), DEHP (HD) (sham: *n*=6; E2: *n*=6), or a mixture of BPA + DEHP (HD) (sham: *n*=6; E2: *n*=7). Data were analyzed by two-way ANOVA, followed by Fisher’s LSD *post hoc* test. ^$^ *p*<0.05, ^$$^ *p*<0.01, ^$$$^ *p*<0.001, comparison between E2-implanted control and EDC-exposed, E2-treated female offspring. **p<0.01, comparison between control-sham and EDC-exposed, sham-implanted offspring. **^++^** p<0.01, comparison between sham- and E2-implanted female offspring of the same EDC treatment group. Error bars represent the standard error of the mean (SEM).

No significant differences were identified in the total exploration of the EPM (**Figure 3A**). An interaction effect (*p* = 0.018; F(5,63)=3) was observed in the number of entries into the open arms (mean ± SEM) (**Figure 3A**). Control-E2 females (5.0 ± 0.3) showed a robust decrease in open-arm entries than their sham counterparts (11.6 ± 0.5; *p* = 0.002), representing an increase in anxiety-like behavior. Interestingly, all of the E2-treated females prenatally exposed to EDCs, with the exception of DEHP (HD)-E2 females, demonstrated anxiolytic effects with significant increases in open-arm entries compared to control-E2 offspring. DEHP (LD)-E2 (*p* = 0.0003) and B+D (HD)-E2 (*p* = 0.002) offspring showed the highest increases in open arm entries relative to their control counterparts at 150% and 120%, respectively.

There were significant EDC effects with regard to the time spent in the center (%; mean ± SEM) of the EPM (p=0.0073; F(5,65)=3.5) (**Figure 3B**). Compared to sham-treated controls (22.8. ± 4.6), the time spent by E2 treated controls (11.9 ± 1.9; p=0.002), E2 treated DEHP (LD) (10.5 ± 1.2; p=0.0003) and E2-treated B+D (LD) (13.4 ± 2.1; p=0.02) groups were significantly lower suggesting increased anxiety in these groups. While the time spent in closed arms would also suggest increased anxiety, there were no significant changes in this parameter.

#### Shock probe defensive burying

**Figure 4:**
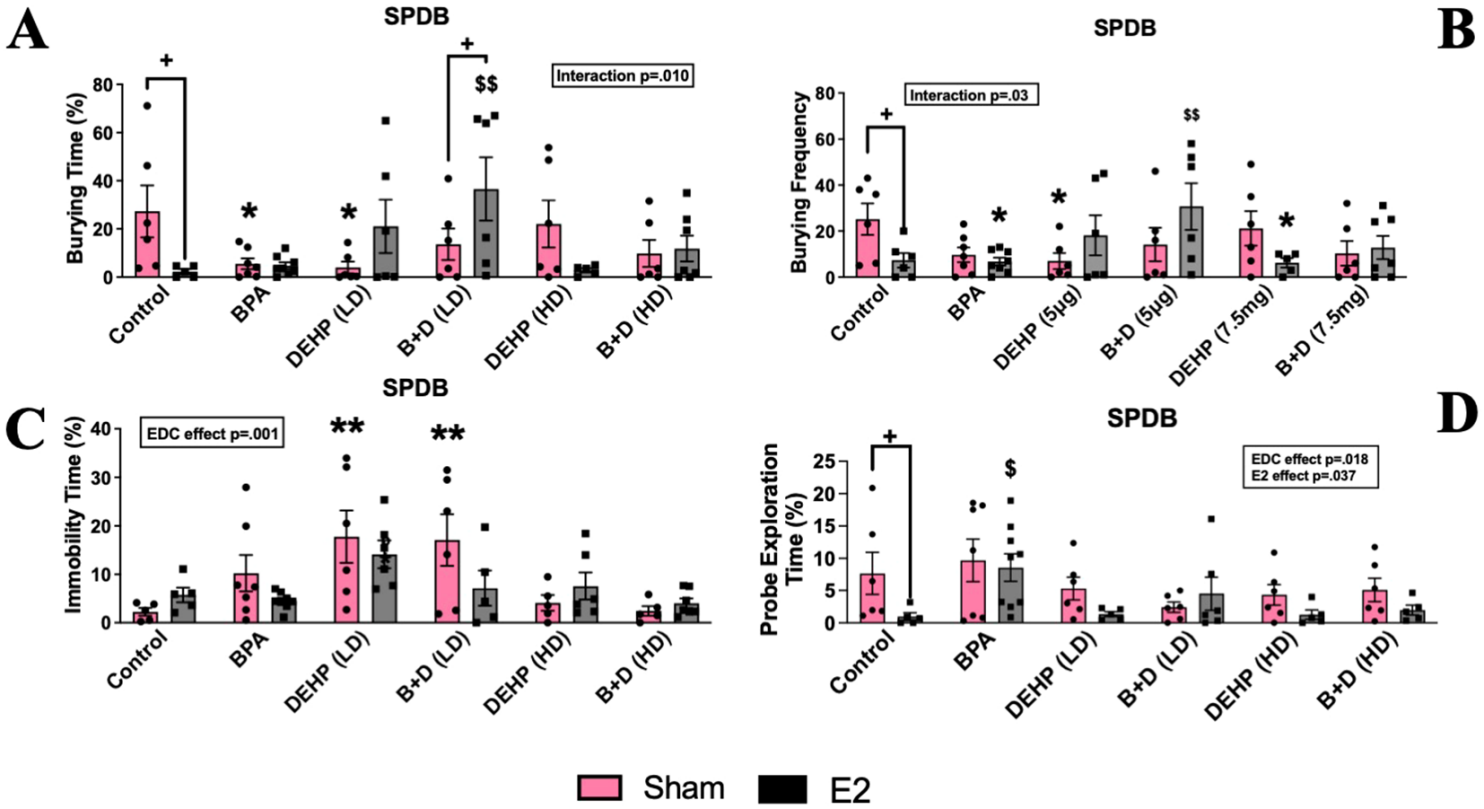
Behavioral effects of prenatal EDC exposure and/or adult E2 treatment in female rat offspring in the shock probe defensive burying (SPDB). **(A)** Amount of time spent burying, **(B)** burying frequency, **(C)** amount of time spent being immobile, and **(D)** amount of time spent exploring the probe. Data were collected from sham- or E2-implanted adult female offspring prenatally exposed to vehicle (Control) (sham: *n*=5-6; E2: *n*=5-7), BPA (sham: *n*=7; E2: *n*=8-9), DEHP (LD) (sham: *n*=6; E2: *n*=5-6), a mixture of BPA + DEHP (LD) (sham: *n*=6; E2: *n*=5-6), DEHP (HD) (sham: *n*=5-6; E2: *n*=5-6), or a mixture of BPA + DEHP (HD) (sham: *n*=5-6; E2: *n*=5-7). Data were analyzed by two-way ANOVA, followed by Fisher’s LSD *post hoc* test. ^*^ *p*<0.05, ^**^ *p*<0.01, comparison between sham-implanted control and EDC-exposed female offspring. ^$^ *p*<0.05, ^$$^ *p*<0.01, comparison between E2-implanted control and EDC-exposed female offspring. **^+^** *p*<0.05, comparison between sham- and E2-implanted female offspring of the same EDC-treatment group. Error bars represent the standard error of the mean (SEM).

There was a significant interaction effect (*p* = 0.010; F(5,62)=3.4) observed in the amount of time spent burying the probe (%, mean ± SEM) (**Figure 4A**) and the burying frequency (mean ± SEM; p=0.03; F(5,63)=2.7; **Figure 4B**). Drastic reductions in burying time were observed in control-E2 (91.5% decrease), BPA-sham (79.7%), and DEHP (LD)-sham (85.2%) offspring compared to control-sham offspring. E2 treatment significantly interfered with burying frequency in control (7.33 ± 3.1), BPA (6.75 ± 1.7) and DEHP (HD) (6.2 ± 2) groups compared to control sham (25.2 ± 6.8; p<0.05). Interestingly, prenatal exposure to B+D (LD) (30.7 ± 10.1) prevented the E2-induced reduction in burying frequency when compared to control (7.33 ± 3.1; p<0.01). This was coupled with significantly decreased bedding height in DEHP (LD)-sham females only (5.9 ± 0.4; *p* = 0.026) relative to their control counterparts (7.8 ± 0.7) (**Table 1**). There was a significant EDC effect in immobility time (p=0.0006; F(5,59)=5.1) with both sham-implanted DEHP (LD) (17.8 ± 5.4; p=0.0011) and B+D (LD) (17.1 ± 5.3; p=0.002) being significantly higher than sham-implanted control (2.2 ± 0.8; **Figure 4C**) indicating increased passive coping in these groups. Significant main effects of EDC (*p* = 0.018; F(5,65)=2) and E2 (*p* = 0.037; F(5,65)=1) were also identified in the amount of time spent exploring the probe (%, mean ± SEM) (**Figure 4D**). E2-exposed control offspring (1.0 ± 0.6; *p* = 0.038) demonstrated a substantial decrease in probe exploration time compared to their sham counterparts (7.7 ± 3.3).

#### Novel object recognition test

**Figure 5:**
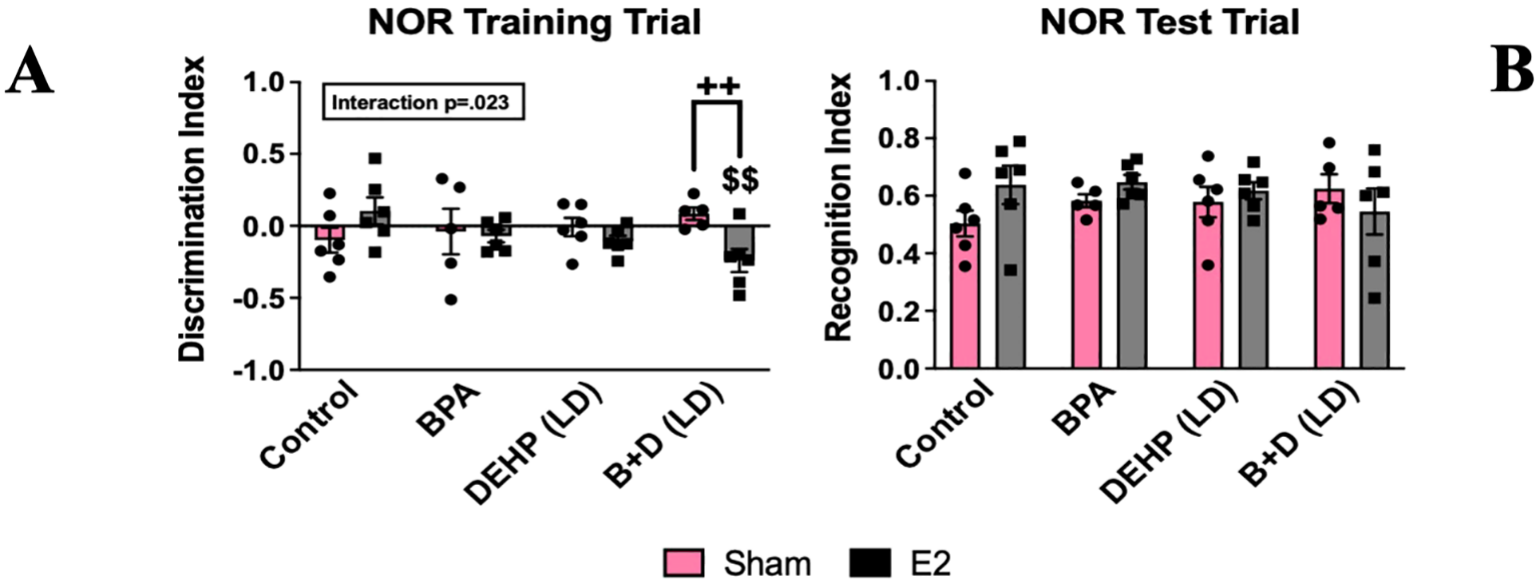
Behavioral effects of prenatal EDC exposure and/or adult E2 treatment in female rat offspring in the novel object recognition test (NOR). **(A)** Discrimination index during the training trial and **(B)** recognition index during the test trial. Data were collected from sham- or E2-implanted adult female offspring prenatally exposed to vehicle (Control) (sham: *n*=6; E2: *n*=6), BPA (sham: *n*=5; E2: *n*=6), DEHP (LD) (sham: *n*=6; E2: *n*=6), or a mixture of BPA + DEHP (LD) (sham: *n*=5; E2: *n*=6). Data were analyzed by two-way ANOVA, followed by Fisher’s LSD *post hoc* test. ^$$^ *p*<0.01, comparison between E2-implanted control and EDC-exposed female offspring. ^++^p<0.01, comparison between sham- and E2-implanted female offspring of the same treatment group. Error bars represent the standard error of the mean (SEM).

A modest interaction effect (*p* = 0.023; F(3,38)=3.6) was observed in the training trial (T1) discrimination index (DI) (mean ± SEM) (**Figure 5A**). B+D (LD)-E2 (-0.2 ± 0.1) females spent significantly more time with the left object compared to control-E2 offspring (0.1 ± 0.1; *p* = 0.004), as well as B+D-sham offspring (0.1 ± 0.0; *p* = 0.009). No differences were observed in the recognition index (**Figure 5B**).

### Effects on hormonal levels

**Figure 6:**
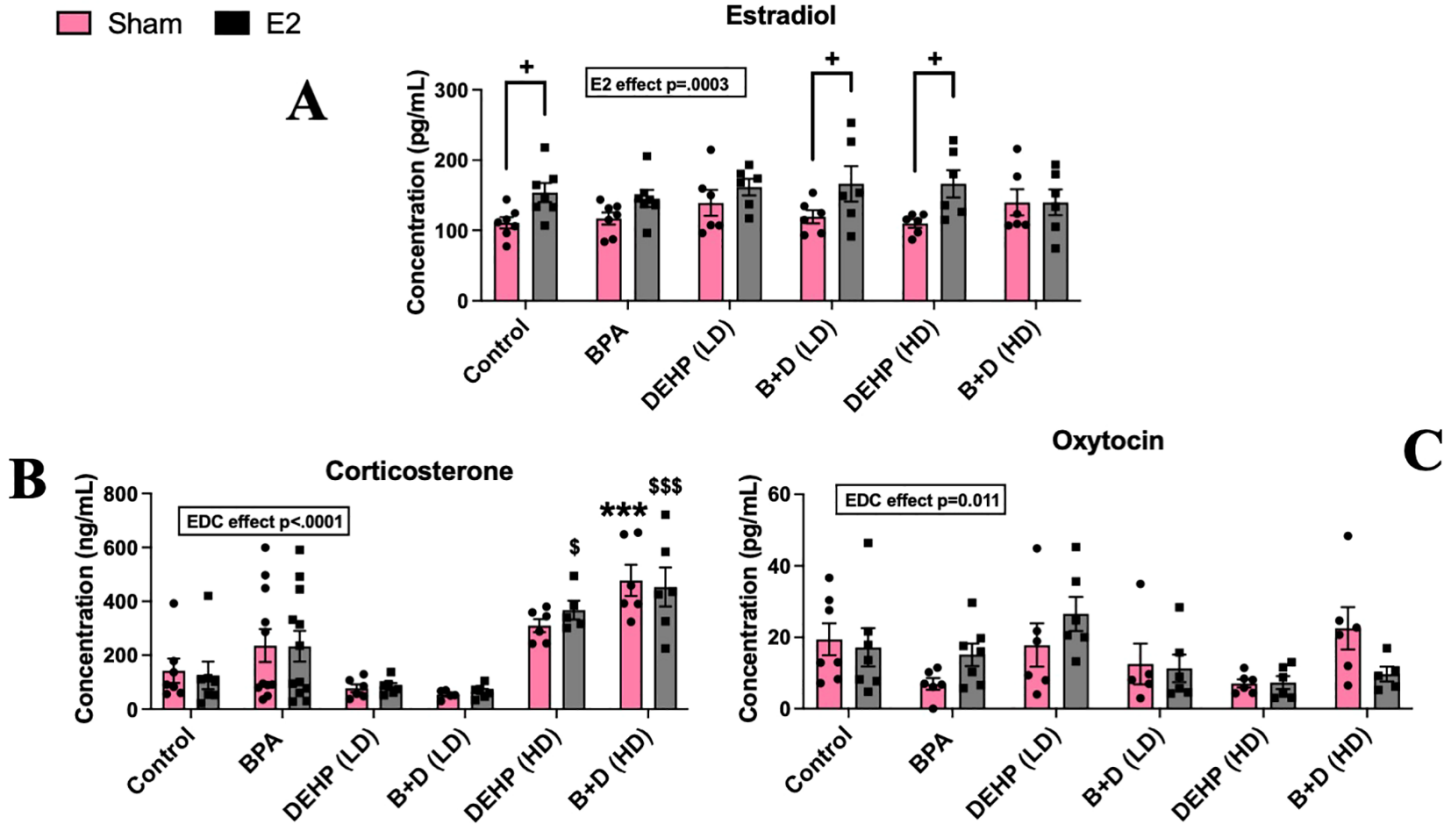
Circulating hormone levels in female rat offspring with prenatal EDC exposure and/or adult E2 treatment. **(A)** Serum estradiol (E2) levels (pg/mL), **(B)** serum corticosterone (CORT) levels (ng/mL), and **(C)** serum oxytocin (OXT) levels (pg/mL). Data were collected from sham- or E2-implanted adult female offspring prenatally exposed to vehicle (Control) (sham: *n*=7; E2: *n*=7), BPA (sham: *n*=6-9; E2: *n*=7-9), DEHP (LD) (sham: *n*=6; E2: *n*=6), a mixture of BPA + DEHP (LD) (sham: *n*=5-6; E2: *n*=6), DEHP (HD) (sham: *n*=6; E2: *n*=5-6), or a mixture of BPA + DEHP (HD) (sham: *n*=6; E2: *n*=5-6). ^***^ *p*<0.001, comparison between sham-implanted control and EDC-exposed female offspring. ^$^ *p*<0.05, ^$$$^ *p*<0.001, comparison between E2-implanted control and EDC-exposed female offspring. ^+^ *p*<0.05, comparison between sham- and E2-implanted female offspring of the same EDC treatment group. Error bars represent the standard error of the mean (SEM).

There was a significant effect of E2 implantation on circulating E2 levels (p=0.0003; F(1,64)=14.81). A significant effect of EDC treatment was also apparent in CORT (p=0.011; F(5,61)=3.287) and OXT levels (p<0.0001; F(5,71)=15.71). E2-treatment significantly increased circulating E2 levels in control animals compared to their sham counterparts (*p* = 0.034) (**Figure 6A**), as expected. In addition, B+D (LD) animals and DEHP (HD)-exposed offspring demonstrated increases in serum E2 levels, compared to their corresponding sham implanted animals (*p <*0.05) (**Figure 6A**).

In contrast, both sham and E2 implanted B+D (HD) offspring had elevated CORT levels (ng/ml; mean ± SEM; 478.6 ± 57.6 and 453.3 ± 72.6 in B+D (HD)-sham and E2 respectively) compared to their corresponding control groups (142.6 ± 44.7 and 125.5 ± 51 in Control-sham and E2 respectively: *p* < 0.001) (**Figure 6B**). However, no changes were observed in OXT levels (**Figure 6C**).

### Effects on brain neurotransmitter activity

#### Paraventricular hypothalamic nucleus

**Figure 7:**
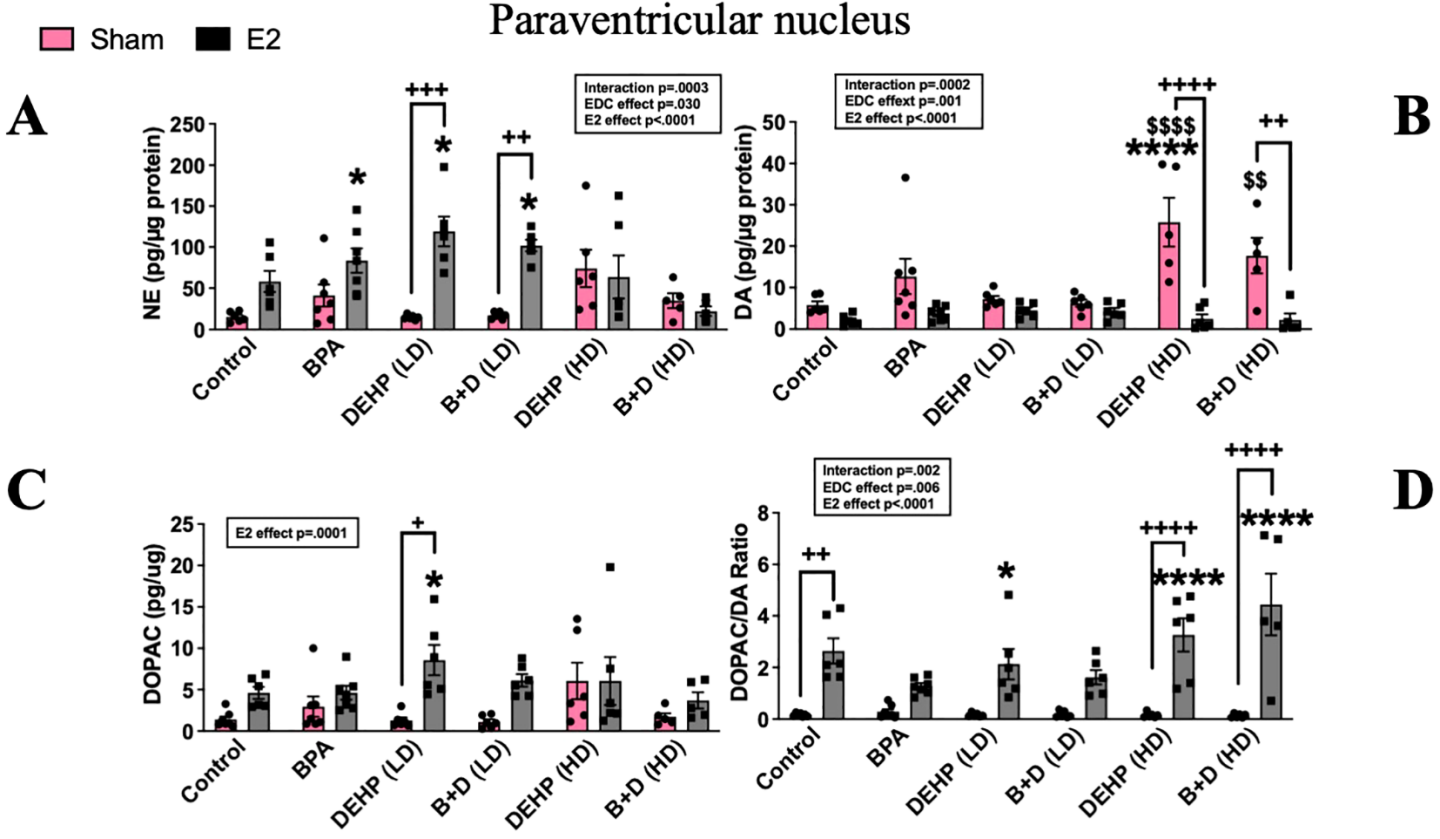
Effects of prenatal EDC exposure and/or adult E2 treatment on monoamine levels and monoamine turnover ratios in the paraventricular nucleus (PVN) of female rat offspring. **(A)** Norepinephrine (NE), **(B)** dopamine (DA), **(C)** serotonin (5-HT) concentrations (mean ± SEM; pg/µg protein), and **(D)** DOPAC/DA ratios (mean ± SEM) in the PVN are shown in the figure. Data were collected from sham- or E2-implanted adult female offspring prenatally exposed to vehicle (Control) (sham: *n*=6-7; E2: *n*=6), BPA (sham: *n*=7; E2: *n*=7), DEHP (LD) (sham: *n*=6; E2: *n*=6), a mixture of BPA + DEHP (LD) (sham: *n*=6; E2: *n*=6), DEHP (HD) (sham: *n*=5-6; E2: *n*=6), or a mixture of BPA + DEHP (HD) (sham: *n*=5-6; E2: *n*=5). ^*^ *p*<0.05, ^****^ *p*<0.0001, comparison between sham-implanted control and EDC-exposed female offspring. ^$^ *p*<0.05, comparison between E2-implanted control and EDC-exposed female offspring. ^+^ *p*<0.05, ^++^ *p*<0.01, ^++++^p<0.0001, comparison between sham- and E2-implanted female offspring of the same EDC treatment group. Error bars represent the standard error of the mean (SEM).

There was a significant effect of prenatal EDC exposure (p=0.029; F(5,60)=2.7), E2 treatment (p<0.0001; F(1,60)=26) and interaction (p=0.0003; F(5,60)=5.5) in terms of NE levels in the PVN. Although there were no differences between EDC-exposed sham animals and the control group, E2-treatment markedly increased NE levels in animals prenatally exposed to BPA (83.6 ± 14.9; p=0.03), DEHP (LD) (119.1 ± 18.1; p=0.0002) and B+D (LD) (101.9 ± 6.9; p=0.003) compared to control sham (15.4 ± 2.5). Moreover, E2 treatment produced a remarkable increase in NE levels in the DEHP (LD) and B+D (LD) groups compared to their corresponding sham-treated groups (14.84 ± 1.1 and 17 ± 1.7 in DEHP (LD) and B+D (LD) groups respectively; p<0.01) (**Figure 7A**).

PVN DA levels (pg/µg protein; mean ± SEM) were markedly elevated in the DEHP (HD) sham (25.8 ± 5.9) group compared to the control sham (5.8 ± 0.8) and control-E2 (2.1 ± 0.5; p<0.0001). DA levels in the B+D (HD) sham group (17.7 ± 4.3) were also elevated compared to control E2 (p<0.01). E2 implantation in these two groups significantly dropped DA levels (**Figure 7B**). DOPAC levels (pg/µg protein; mean ± SEM) in the PVN were not affected to a large extent. There was a modest increase in DOPAC levels in the DEHP (LD)-E2 group (8.57 ± 1.8) compared to the corresponding sham (1.28 ± 0.3) and the control sham group (1.38 ± 0.3; p<0.5) (**Figure 7C**). The ratio of DOPAC/DA was significantly altered with EDC treatment (p=0.0055; F(5,62)=3.69), E2 implantation (p<0.0001; F(1,62)=103.5) and their interaction (p=0.002; F(5,62)=4.32). E2 implantation increased DOPAC/DA ratio in controls (2.64 ± 0.49), DEHP (HD) (3.27 ± 0.6) and B+D (HD) (4.45 ± 1.2) groups compared to the corresponding sham -implanted rats (0.17 ± 0.02, 0.15 ± 0.04 and 0.129 ± 0.03 in control, DEHP (HD) and B+D (HD) respectively. There was also a dose-dependent increase in DOPAC/DA ratio in DEHP-E2 rats compared to the control sham group (**Figure 7D**).

B+D offspring generally seemed to mirror the effects observed in their low- or high-dose DEHP counterparts in the PVN. For example, B+D (HD)-sham offspring had significantly higher PVN DA levels (*p* = 0.017) (**Figure 7B**) compared to control-sham females, resembling DEHP (HD)-sham offspring. In 5-HT levels (**Table 2**), there was a modest E2 and interaction effect (p<0.05); however *post-hoc* tests did not reveal any significant changes between groups.

**Table 2:** Neurotransmitter data of sham and E2-treated female offspring following low-dose (5 µg) and high-dose (7.5 mg) prenatal EDC exposure.

Neurotransmitter	Control		BPA (5 µg)		DEHP (5 µg)		BPA + 5 µg DEHP		DEHP (7.5 mg)		BPA + 7.5 mg DEHP	
	Sham	E2	Sham	E2	Sham	E2	Sham	E2	Sham	E2	Sham	E2
Paraventricular Nucleus												
*5-HT (pg/µg)*	2.2 ± 0.4	10.3 ± 1.5	3.5 ± 0.7	6.8 ± 1.2	4.1 ± 0.4	7.7 ± 0.8	3.3 ± 0.5	5.6 ± 1.6	6.9 ± 2.5	8.6 ± 3.8	7.9 ± 2.8	3.5 ± 1.6
*5-HIAA (pg/*µ*g)*	11.0 ± 1.8	36.9 ± 6.5	24.4 ± 6.2	20.3 ± 4.4	15.9 ± 1.4	26.2 ± 4.2	18.2 ± 1.0	17.7 ± 2.4	103.6 ± 42.4^**^ **^,+^**	28.3 ± 11.6**^+^**	63.0 ± 30.2	15.7 ± 4.2
*5-HIAA/5-HT ratio*	5.7 ± 0.9	3.7 ± 0.4	7.9 ± 2.3	3.0 ± 0.3	4.1 ± 0.6	3.6 ± 0.7	6.0 ± 0.7	3.4 ± 0.4	17.3 ± 3.7^****^ **^,++++,^** **^$$$$^**	4.1 ± 0.8**^++++^**	7.0 ± 0.8	6.4 ± 1.2
Hippocampus												
*5-HT (pg/*µ*g)*	2.0 ± 0.6	2.3 ± 0.3	1.8 ± 0.3	1.8 ± 0.3	2.4 ± 0.3	1.5 ± 0.3	2.7 ± 0.6	1.8 ± 0.4	1.4 ± 0.2	2.7 ± 0.6	1.3 ± 0.2	2.1 ± 0.5
*5-HIAA (pg/*µ*g)*	8.1 ± 2.6	12.3 ± 1.0	9.5 ± 1.3	11.2 ± 1.8	10.0 ± 0.6	9.3 ± 1.3	11.8 ± 2.1	10.6 ± 1.1	13 ± 1.3	15.7 ± 1.6	10.7 ± 2.4	14.4 ± 2.4
*5-HIAA/5-HT ratio*	4.0 ± 1.0	5.8 ± 0.6	7.4 ± 2.3	6.6 ± 0.9	4.5 ± 0.6	7.1 ± 1.4	4.9 ± 0.8	7.1 ± 1.4	10.5 ± 2.6	6.8 ± 1.1	9.1 ± 1.5	8.2 ± 1.9

EDC, endocrine-disrupting chemicals; E2, estradiol; BPA, bisphenol A; DEHP, di-(2-ethylhexyl) phthalate. Data are presented as mean ± SEM and analyzed by two-way ANOVA followed by Tukey's post hoc test. *** p < 0.001, **** p < 0.0001, significant difference between sham-implanted Control and EDC females, $$$$ p<0.0001, significant difference between Control E2 treated animals and the EDC females; + p < 0.05, ++ p < 0.01, +++ p < 0.001, ++++ p < 0.0001, significant difference between sham and E2-treated females of the same treatment group.

#### Hippocampus

**Figure 8:**
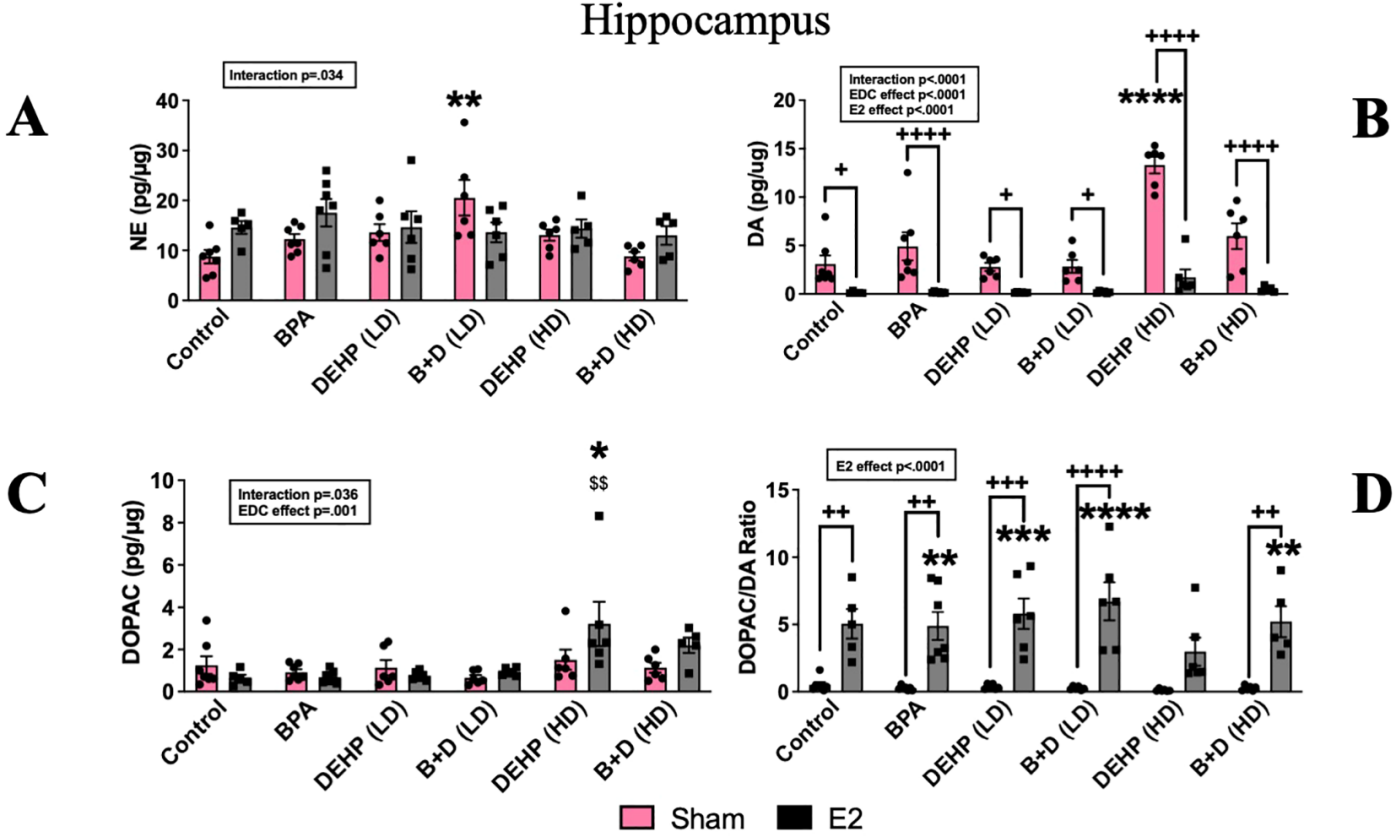
Effects of prenatal EDC exposure and/or adult E2 treatment on monoamine and metabolite levels and monoamine turnover ratios in the hippocampus (HC) of female rat offspring. **(A)** Norepinephrine (NE), **(B)** dopamine (DA), **(C)** 3,4-dihydroxyphenylacetic acid (DOPAC) concentrations (mean ± SEM; pg/µg protein), and **(D)** DOPAC/DA ratios (mean ± SEM) in the HC are shown in the figure. Data were collected from sham- or E2-implanted adult female offspring prenatally exposed to vehicle (Control) (sham: *n*=7; E2: *n*=5), BPA (sham: *n*=7; E2: *n*=7), DEHP (LD) (sham: *n*=6; E2: *n*=6), a mixture of BPA + DEHP (LD) (sham: *n*=6; E2: *n*=6), DEHP (HD) (sham: *n*=6; E2: *n*=5-6), or a mixture of BPA + DEHP (HD) (sham: *n*=6; E2: *n*=5). ^**^ *p*<0.01, ^***^ *p*<0.001, ^****^ *p*<0.0001, comparison between sham-implanted control and EDC-exposed female offspring. ^$$^ *p*<0.01, comparison between E2-implanted control and EDC-exposed female offspring. ^+^ *p*<0.05, ^++^ *p*<0.01, ^+++^p<0.001, ^++++^p<0.0001, comparison between sham- and E2-implanted female offspring of the same treatment group. Error bars represent the standard error of the mean (SEM). Indicates p<0.05 difference between sham and E2 implanted rats.

Monoamine and metabolite levels within the HC are provided in **Figure 8**. There was a modest effect of interaction (p=0.033; F(5,62)=2.6 in terms of NE levels in the HC. NE levels (pg/µg protein; mean ± SEM) in the B+D (LD) sham group (20.5 ± 3.6) was significantly higher than the control-sham group (8.75 ± 1.3; p<0.01) (**Figure 8A**).

There was a significant impact of EDC exposure (p<0.0001; F(5,61)=16.9), E2 treatment (p<0.0001; F(1,61)=117.7) and interaction (p<0.0001; F(5,61)=9.19). DA levels (pg/µg protein; mean ± SEM) in the HC of DEHP (HD)-sham offspring (13.3 ± 0.8) were significantly higher than the rest of the groups. The most pronounced differences were apparent from control-sham (3.09 ± 0.8) and DEHP (HD)-E2 (1.73 ± 0.8; *p* < 0.0001). Treatment with E2 also significantly reduced DA levels across the board (p<0.05 to p<0.0001) (**Figure 8B**). In contrast to DA levels, ANOVA revealed a modest interaction (p=0.036) and a strong EDC effect on DOPAC levels (p=0.001; F(5,61)=5.07). DOPAC levels (pg/µg protein; mean ± SEM) in the DEHP (HD)-E2 group alone were significantly higher (3.2 ± 1) compared to control-E2 (0.6 ± 0.1; p=0.003) and control sham (1.26 ± 0.4; p<0.05) (**Figure 8C**). Corresponding to the changes in DA and DOPAC levels, ANOVA revealed a significant impact of E2 treatment on DOAPC/DA ratio (p<0.0001; F(1,61)=111.2. Almost all E2-treated groups (except for the DEHP-HD group) had higher DOPAC/DA ratios compared to the control sham group (p<0.01 to p<0.0001). In addition, the E2 treated BPA, DEHP (LD), B+D (LD) and the B+D (HD) groups were significantly higher than their corresponding sham implanted groups (p<0.01 to p<0.0001).

Serotonergic activity was entirely unaffected in the HC (**Table 2**).

## Discussion

Results from this study demonstrate that prenatal exposure to low doses of BPA with increasing doses of DEHP alone or in combination can impact behavioral outcomes. When challenged with chronic exposure to low-dose E2 in adulthood, some effects were exacerbated, while others were negated. These outcomes are specific to the EDC used and the parameter measured. Each behavioral paradigm employed in this study evaluates distinct aspects of stress-related behaviors, with the OFT targeting exploratory activity and novelty exposure (38). The control offspring did not demonstrate any changes in OFT behavior as a result of E2 treatment. Yet, E2 treatment in offspring with prenatal exposure to BPA, DEHP, or a combination of BPA + DEHP (HD) elevated anxiety-like behavior in this test. While a plethora of studies exist in the literature illustrating alterations in anxiety-like behavior in females with developmental BPA (39–42) or DEHP (43–46) exposure, ours is the first study to incorporate a dual exposure paradigm evaluating the cumulative behavioral effects of prenatal EDC exposure followed by adult E2 treatment. Importantly, we establish that E2 treatment in adult offspring that were prenatally exposed to a mixture of BPA and DEHP can also lead to anxiogenic effects.

While the OFT assesses exploration and novelty exposure, the EPM evaluates unconditioned anxiety (38). Treatment with E2 in control offspring increased anxiety-like behavior in the EPM. This result is partially in accordance with a prior study from our lab, in which adult female rats were treated with E2 at the same dose and duration. E2-treated rats were found to exhibit increased anxiety-like behavior in that study as well, but in the OFT rather than the EPM (23). Present findings are also in line with studies that have demonstrated elevated anxiety-like behavior in response to E2 in rodents (34, 47, 48), but contrast with a number of studies that have found the opposite (34, 49, 50).

Nevertheless, inconsistencies in anxiety-like behaviors across various behavioral paradigms have been reported before (51, 52). The results from our study show that chronic treatment for 90 days with 20 ng/day of E2 increases unconditioned anxiety in healthy adult female rats. More importantly, E2 treatment reversed this effect in EDC-exposed offspring, with the exception of DEHP (HD) females, and reduced anxiety-like behavior in these offspring. This is in complete contrast to the effects observed in control offspring, implying that prenatal EDC exposure probably induces subtle changes in the brain that drastically alter the anxiety response when animals are treated with E2 in adulthood.

The results from control offspring in this test were particularly striking because they demonstrate that E2 treatment reduces active coping mechanisms in healthy females; specifically, these offspring show decreased preference for burying and probe exploration in the SPDB test. This finding contradicts other studies that have found a positive correlation between E2 administration and active coping behaviors (53, 54), or no correlation (55). Nevertheless, several factors differ between our study and the aforementioned studies, including presence or absence of ovaries, dose of E2, and route of E2 administration. While control-E2 offspring had reduced probe exploration time, BPA-E2 offspring spent significantly more time exploring the probe than their control counterparts, indicative of risky and inappropriate responses to an aversive stimulus. Burying in this test is often interpreted as an active, adaptive coping style (56); therefore, these findings imply that prenatal EDC exposure may inhibit this adaptive behavior possibly making these offspring more prone to stress. In support of this theory, these offspring also had increased open arm entries in the EPM, which is relatively more stressful and riskier compared to the OFT (57). Research is lacking into the effects of BPA on risk-taking, but E2 has been linked with increased risk-taking behaviors (58, 59). Overall, we can conclude that BPA exposure alone and in combination with adult E2 alters stress-related behaviors and may induce inappropriate responses to stress. In contrast to BPA, B+D (LD)-E2 animals spent considerably more time burying compared to their control and B+D (LD)-sham counterparts.

In contrast to probe exploration and burying, animals exposed prenatally to DEHP (LD) and B+D(LD) that were sham implanted had substantial increases in immobility time compared to control-sham, representing a shift to passive, maladaptive coping styles (56). The results from the B+D (LD) females were especially intriguing, since sham offspring preferred passive coping strategies, whereas their E2-treated counterparts engaged in active coping. This is significant because B+D (LD) offspring showed a robust reversal of behavioral effects in this test relative to control offspring. We can conclude that chronic E2 treatment in female offspring with prenatal B+D exposure at the low dose leads to aberrant defensive behaviors.

Even though we did not observe any significant differences in cognition in the NOR in controls, a near-significant increase in the recognition index (*p* = 0.067) was, in fact identified in E2-treated offspring. In other words, E2-treated controls showed a trend for enhanced object recognition. Therefore, E2 appears to have a positive correlation with cognition and may boost cognitive abilities in adult females, which is consistent with previous studies (34, 50, 60). It should be noted that an increase in sample size in our study may have produced statistically significant results. Interestingly, the pattern of novel object exploration in B+D (LD) females was reversed compared to that of control offspring, which is indicative of atypical exploratory behavior. This also demonstrates that E2 treatment led to aberrant object exploration in B+D (LD) offspring without impacting object recognition. This again suggests that early exposure to even low doses of B+D possibly produces changes in the neurocircuitry that become apparent when animals are exposed to E2 in adulthood.

Findings from circulating hormone levels indicate that CORT levels were unaffected by E2 implantation in controls. Considering that estradiol can influence hypothalamic-pituitary-adrenal (HPA) axis activity in an estrogen receptor subtype-dependent manner (61), as well as increase plasma OXT levels (62), these results were surprising. We can conclude that chronic E2 treatment may be linked with the adverse behavioral effects we observed in control females.

However, CORT levels were markedly increased in B+D (HD) sham and E2 implanted females. This finding is consistent with the OFT results, in which B+D (HD)-E2 females demonstrated increased anxiety-like behavior. Increased CORT may be an underpinning source of the anxiogenic effect. Overall, the hormonal findings from this study are significant because the effects of E2 implantation on circulating E2 levels were abolished in many of the EDC groups, and the CORT results imply a hyperactivity of the HPA axis, specifically in offspring with high-dose EDC exposures.

In rodents, OXT administration is associated with anxiolytic effects and suppressed HPA axis activation (63, 64); similar effects are also observed in humans (65). The results from the OXT analyses in the current study appear to support this and correspond with the behavioral effects observed in E2-treated DEHP females. This indicates that E2 treatment may interact with OXT in the body to mediate anxiety-like behavior, particularly in DEHP-exposed females. The reasons pertaining to a lack of changes in E2 levels in the high-dose B+D group are unclear. It is possible that treatment with a combination of BPA and DEHP (HD) desensitizes offspring to the effects of E2. Specifically, BPA may counteract the effects of DEHP (HD) on circulating E2, since only DEHP (HD)-E2 offspring showed increased serum E2.

NE levels in the PVN and CORT together represent HPA axis activation and are directly correlated, as NE infusions directly into the PVN have been shown to increase circulating CORT (66). Yet, results from the present study revealed a dysregulation of the stress axis. While E2 implantation increased PVN NE levels in the control, BPA, and low-dose EDC groups, none of these groups showed changes in CORT. This could suggest a downregulation of adrenergic receptors in the PVN or a epigenetic modification of the receptors that could impact their function or an adaptive response to chronic E2 exposure. On the contrary, only the high-dose DEHP groups showed significant increases in CORT; however, these groups did not exhibit corresponding elevations of PVN NE. This might indicate a direct impact on adrenal activity, however, there is evidence to suggest that DEHP exposure early on, could decrease CORT secretion by inhibiting the rate-limiting enzyme in steroid synthesis in the adrenal (67).

Besides NE, DA levels in the PVN were increased in sham-implanted BPA, DEHP (HD) and B+D (HD) groups compared to their E2-treated counterparts. Paralelling these findings, The DOPAC/DA ratio was increased in all E2-treated offspring, except the group exposed to BPA. This suggests increased DA metabolism in these groups. The abolishment of an E2 effect in BPA offspring is intriguing because it suggests that these offspring may be desensitized to the effects of E2 on DA metabolism in the PVN. BPA is known to bind to estrogen receptors and induce estrogenic effects (68), although it can also serve as an antiestrogen by competing with endogenous E2 to block estrogenic responses (69, 70). Correspondingly, BPA is also capable of inhibiting or antagonizing estrogenic activity within the brain when co-administered with E2 (71, 72). Hence, it appears that adult offspring with prenatal BPA exposure may show some concerning effects on dopaminergic activity in the PVN, especially when combined with adult exogenous E2.

B+D offspring generally mirrored the effects observed in the low-dose and high-dose DEHP counterparts in the PVN. These results are in agreement with our previous findings in male rats where the same dose of BPA and DEHP reduced DA levels in the PVN, and this was accompanied by a decrease in probe burying time in the SPDB (73).

The increase in HC NE levels in the B+D (LD) group correlates well with the enhanced center zone exploration in the OFT. HC NE is released following novelty exposure and arousal, activating the locus coeruleus-noradrenergic system (74). It is possible that NE levels were elevated in the HC in response to novelty, which mediated the behavioral effects observed in these offspring. Following NE, DA levels in the HC were also elevated in the DEHP (HD) group in the present study. Interestingly, E2 implantation in all the EDC groups was able to drive DA levels down. This was accompanied by an increase in the DOPAC/DA ratios suggesting that the increased metabolism of DA was probably the reason for the reduction in DA levels. Since DOPAC is a major metabolite of DA and is formed as a result of DA catalysis (75), elevated hippocampal DA metabolism and turnover may be an underlying mediator of the increased anxiety-like behavior found in DEHP (HD) female offspring with E2 treatment. Overall, it is important to highlight that exposure to different doses of DEHP can lead to drastically distinct outcomes on behavior, hormones, and brain monoamines.

As mentioned earlier, E2 treatment in control females increased unconditioned anxiety in our study. This is partially consistent with a previous study from our lab, which discovered anxiogenic behavioral effects accompanied by reduced DA levels after E2 treatment, but only in the central amygdala and not in the HC (23). Nevertheless, the HC belongs to the limbic system along with the amygdala (76), and our findings demonstrate that enhanced dopaminergic functioning in limbic regions may be necessary to reduce anxiety-like behaviors. Finally, all sham-implanted offspring in vehicle and EDC-exposed groups had higher DA levels in the HC than their E2-treated counterparts. This sensitization to E2 is probably due to an EDC-induced effect and may involve enhanced expression of estrogen receptors or epigenetic modifications of these receptors and needs additional investigation.

### Environmental implications

Our study incorporates environmental exposures that are ubiquitous and inevitable in everyday life – environmentally relevant doses of EDCs, both independently and in combinations, as well as chronic E2 treatment. Our findings determine that healthy control females treated with chronic E2 in adulthood show increased unconditioned anxiety and engage less in active coping strategies. In contrast, E2 treatment in prenatally EDC-exposed females often reverses or abolishes neurobehavioral effects, implying that EDCs interact with E2 to alter behavioral endpoints. E2-treated offspring exposed to BPA combined with the low dose of DEHP, in particular, show distinctive behavioral effects relative to other EDCs. In conclusion, this study provides evidence that prenatal EDC exposures and adult exogenous E2 treatment cumulatively alter behavior, hormones, and brain monoamines in a dose-dependent manner. This calls for additional review and modifications of current regulatory practices regarding harmful EDC exposures, and broadens the knowledge on chronic estradiol exposures in adult females.

## Data Availability

The original contributions presented in the study are included in the article/supplementary material. Further inquiries can be directed to the corresponding author.
